# Temperature and microclimate refugia use influence migratory timings of a threatened grassland bird

**DOI:** 10.1186/s40462-023-00437-7

**Published:** 2023-12-01

**Authors:** Rita F. Ramos, Aldina M.A. Franco, James J. Gilroy, João P. Silva

**Affiliations:** 1 CIBIO/InBIO, Centro de Investigação em Biodiversidade e Recursos Genéticos, Laboratório Associado, Universidade do Porto, Campus Agrário de Vairão, Vairão, 4485-661 Portugal; 2 CIBIO/InBIO, Centro de Investigação em Biodiversidade e Recursos Genéticos, Laboratório Associado, Instituto Superior de Agronomia, Universidade de Lisboa, Tapada da Ajuda, Lisboa, 1349-017 Portugal; 3 Departamento Biologia, Faculdade de Ciências, Universidade do Porto, Porto, Portugal; 4 BIOPOLIS Program in Genomics, Biodiversity and Land Planning, CIBIO, Campus de Vairão, Vairão, 4485-661 Portugal; 5 School of Environmental Sciences, University of East Anglia, Norwich, UK; 6 Estação Biológica de Mértola, Mértola, Portugal

**Keywords:** Partial migration, Migratory behaviour, Individual repeatability, Population variability, Birds, Environmental cues

## Abstract

**Background:**

Seasonal changes in resource availability are known to influence the migratory behaviour of animals, including both timing and distance. While the influence of environmental cues on migratory behaviour has been widely studied at the population level, it has rarely been examined at the spatial scale at which individuals experience their environment. Here, we test the hypothesis that individuals exposed to similar large-scale environmental cues may vary in migratory behaviour in response to the different microclimate conditions they experience at fine scales.

**Methods:**

We combine high-spatial and temporal resolution microclimate and habitat information with GPS tracking data for a partially migratory threatened grassland bird. Data from 47 little bustards (*Tetrax tetrax*; 67 breeding events) tracked between 2009 and 2019 was used to (i) evaluate individual consistency in migratory behaviour (timing and distance) and (ii) assess whether the local environmental characteristics experienced by individuals – and in particular their use of microclimate refugia - influence distance and timing of migration, from and to the breeding sites.

**Results:**

Migratory distance was consistent for birds tracked over multiple years, while the timing of migration showed high variability among individuals. Departures from breeding areas spanned from May to August, with a few birds remaining in their breeding areas. Vegetation greenness (a proxy for food availability) was positively associated with the time birds spent in the breeding area. The best model also included a positive effect of microclimate refugia availability on breeding season length, although an interaction with temperature suggested that this effect did not occur at the highest relative temperatures. The return date to breeding grounds, although spanning from September to April, was not influenced by the environmental conditions or food availability.

**Conclusions:**

Food availability, measured by a vegetation greenness proxy, was associated with later migration at the end of the breeding season. Availability of cooler microclimate refugia may also allow for later departures from the breeding sites in all but the hottest conditions. Management measures that increase microclimate refugia availability and provide foraging resources can thus potentially increase the length of the breeding season for this species.

**Supplementary Information:**

The online version contains supplementary material available at 10.1186/s40462-023-00437-7.

## Background

Migration is a complex behaviour undertaken by billions of organisms annually. These seasonal movements are primarily associated with declines in food availability and deterioration of environmental conditions [1]. The decision to migrate, however, may be influenced by internal factors such as experience or physiological condition, or external factors like high temperatures [1, 2]. Adjusting the timing of migration allows individuals to avoid spatiotemporally unsuitable environments, increasing survival and fitness [3, 4].

Migratory species can vary from fully obligate migrants, where all individuals undertake seasonal movements between distinct geographical sites [3], to partial-migrants where a proportion migrate while others remain resident at their breeding sites [1, 4, 5]. Within a species distribution, environmental variability can affect the frequency of migratory individuals within a population [6]. Individuals may also perform short-, medium- or long-distance seasonal migrations, to one or several destinations, across environmental gradients [4, 5] and sex and age are known to influence the migration strategy individuals adopt [7, 8]. Partial migration is more common than previously thought [9] and likely to be maintained if both migratory behaviours (residency and migration) yield equivalent fitness, or each confers different benefits to individuals [9–11]. This migratory diversity has been associated with greater population-scale resilience to environmental changes in breeding and post-breeding areas [12].

Changes in environmental conditions can lead to variability in the timing of migration, breeding and even moulting [13–15]. For example, precipitation and temperature at breeding sites have been shown to influence the departure from the breeding area (i.e. the start of autumn migration) for four trans-Saharan and six intra-European passerine species that migrate through Heligoland, Germany [16].

Migratory repeatability - i.e. whether individuals perform similar migrations between years – is a good indicator of the extent to which individual migratory decisions are shaped by responses to environmental cues [17]. Common terns (*Sterna hirundo*) breeding in northwest Germany, for example, showed high within-individual repeatability in most aspects of their migratory journeys, suggesting a relatively limited impact of environmental cues on their migratory decisions [18]. While elk (*Cervus elaphus*) in Canada, by contrast, showed low individual repeatability and often changed between resident and migratory strategies [19]. European shags (*Phalacrocorax aristotelis*), a partially migratory species breeding in Scotland, 64% of the individuals kept their migratory strategy (resident, early or late-migrant), between years [17], translating to a relatively high within-individual repeatability. However, like migratory strategy, within-individual repeatability can be influenced by sex and age [20].

Despite the increasing body of literature on migratory movements of partial migratory species, our understanding of how environmental conditions influence migratory responses is still limited.

The factors that influence between-individual variability in migratory parameters within populations have been examined at broad spatial scales [17, 21, 22], contributing to a general understanding of individual responses to environmental variability. However, there is a mismatch between the macro spatial scale most studies use to quantify environmental variation and the fine spatial scale at which individuals experience their environment [23]. Fine-scale heterogeneity in environmental conditions allows for local areas with cooler temperatures than surrounding conditions (hereafter microrefugia); these may provide opportunities for individuals to persist in regions where larger-scale climate conditions become unsuitable [24–26]. Potentially, migratory patterns may also be influenced by microrefugia, particularly for species that are highly sensitive to temperatures, but this remains to be shown. Access to microrefugia is an increasing focus of ecological studies [27, 28], as it can increase individuals’ fitness and can help predict species responses to environmental change [25].

Understanding species’ responses to microclimate conditions is data-demanding and logistically challenging due to the need to combine animal movement and environmental data. In recent decades, high resolution GPS tracking devices have allowed scientists to study animal movement, behaviour, and habitat use at high spatial and temporal resolution scales [29, 30], but availability of environmental data matching the temporal and spatial resolutions experienced by organisms has been limited [26, 31, 32]. Here we combine high resolution animal tracking data with newly developed microclimate modelling tools to determine the influence of environmental conditions on individual migratory decisions. We use GPS tracking data of a grassland bird from a long-term study, to (i) evaluate individual consistency in migratory timings (of departure and return) and distance travelled, and to (ii) evaluate the influence of microclimate refugia, alongside other environmental characteristics, as determinants of variability in migration.

## Methods

### Study area and study system

The Iberian Peninsula is simultaneously a global biodiversity hotspot [33] and one of the world’s most vulnerable regions to climate change [34]. The region is expected to suffer from extensive warming and increasing drought frequency in the near future [35], which are predicted to lead to species range contractions [34]. Species inhabiting semi-natural grasslands, with flat open areas and low vegetation cover, are particularly exposed to high temperatures throughout the year.

The little bustard, *Tetrax tetrax* (Linnaeus, 1758), is a medium-sized grassland specialist bird classified globally as ‘Near Threatened’ [36]. In the Iberian Peninsula, the species is partially migratory, with migratory individuals performing short- (mean ≈ 20km) to medium-distance (mean ≈ 400km) movements [37]. Migration takes place at the end of the breeding season (between May and August) when temperatures increase and vegetation starts to dry, limiting trophic resources [38]. Despite recent severe population declines in both Portugal and Spain due to habitat loss and degradation [37, 39], which may be exacerbated by climate change [40, 41], Iberia is still home to the most significant little bustard breeding populations in Western Europe.

### Satellite GPS tracking data

Between 2009 and 2019, 77 male little bustards were captured and tagged in five breeding areas across the Southwestern Iberian Peninsula, in Alentejo (Portugal) and Extremadura (Spain), during the breeding season (April and May). Little bustards breed in an exploded lekking system [42], where breeding males defend their territories from other males and show exuberant displaying behaviour to attract visiting females [42]. Breeding males were captured using a decoy (stuffed female) and snares [26, 43]. Females, on the other hand, are extremely difficult to capture.

GPS tracking devices, which varied between 2% and 4% ($\stackrel{-}{x}$ = 3.2%) of the birds’ body mass [44], were deployed using a thoracic harness made of Teflon Ribbon with a weak link to avoid lifelong deployment. Two types of Solar GPS devices were used: Platform Transmitter Terminal (Solar Argos/GPS 30 g PTT - Microwave Telemetry), deployed on 19 birds between 2009 and 2011, and 28 Global System for Mobile Communications (GSM) devices (Flyway 25 g - Movetech Telemetry), deployed between 2014 and 2019. Transmitters were programmed to record a GPS position every 2 h (PPT) or 10 to 30 min (GSM). Bird trapping and GPS tagging were approved by the Instituto da Conservação da Natureza e das Florestas (Portuguese authority), through licenses to João Paulo Silva (ICNF/CAPT/2014, ICNF/CAPT/2015), and Consejería de Medio Ambiente y Rural, Políticas Agrarias y Territorio of the Junta de Extremadura (Spanish authority), through the license to José Mª Abad-Gómez.

We only utilised information from birds captured before the 1st of May, which had at least seven days of associated data before departing from the breeding area, leaving 47 birds for analysis. Restricting our sample to birds caught before 1st May also ensures we fully sample the period during which birds are typically more vulnerable to increasing temperatures and food shortages [45, 46].

### Migration timings and distance

The 47 birds provided information for 67 breeding seasons, with 12 and 4 birds being followed during two and three consecutive breeding seasons, respectively. For each bird-season we identified the date of departure from the breeding area, defined as movement away from the centroid of the breeding locations for a minimum period of one month. Birds that continued to use the breeding area throughout the year, even after the breeding season had ended, were referred to as residents [37].

For migratory birds, we determined, when possible, the date birds returned to the breeding areas. For some birds, it was not possible to obtain a return date, either because the bird died, or the tracking device failed.

Each bird’s daily centroid coordinates were calculated using QGIS version 3.10 [47], for both breeding and post-breeding seasons. Subsequently, the mean centroid of the breeding season was retrieved for each individual. The total distance travelled by each bird was determined using the cumulative sum of the distance between that mean centroid and the daily post-breeding centroids.

### Environmental cues

For the breeding season, environmental variables were collected between the 1st of May and the departure date of each bird. Temperature was modelled at fine spatial scales using the *microclima* [48] and *NicheMapR* [49] packages in R version 4.1.0 software [50]. We generated hourly temperatures modelled at a 30 × 30 m resolution calculated at 20 cm above the ground [26]. This spatial resolution is likely to miss some small microclimate refugia features but is the best possible resolution considering the current land cover data availability [26].

We obtained the hourly temperature for each GPS location and the minimum and median temperature within a 500 m buffer of the birds’ locations [26]. All temperature variables were then averaged by day, to minimize the differences in programming between the tagging devices.

Little bustard breeding season spans from April to June [51], though some individuals remain in the breeding area until August. During this period, temperatures can range between 20 and 45ºC. We quantified temperature exposure for each individual relative to the population average using a Generalised Addictive Model (GAM), from the *mgcv* [52] R package, fitting a Gaussian regression smooth to estimate mean daily temperatures of the studied population as a function of Julian date. We summed the residuals of each bird’s daily temperature exposure from May 1st until the bird’s departure date as an index of each individual’s overall exposure to higher or lower temperatures, relative to the studied population within each bird’s tracking period (hereafter ‘relative temperature exposure’).

For each little bustard GPS location, the availability of microclimate refugia was defined as the presence of areas with minimum temperatures at least 0.5ºC cooler than the median temperature within 500 m surrounding each GPS location [26]. We then calculated the percentage of GPS locations with available microclimate refugia (1) throughout each bird’s breeding season.

Satellite-derived Normalized Vegetation Index (NDVI) is a measure of vegetation greenness and biomass [53]. Since little bustards feed mainly on green plants, NDVI can be used as predictor of food availability [53]. We obtained NDVI values for all little bustard GPS locations using 8-day composite 250 m spatial resolution MODIS (Moderate Resolution Imaging Spectroradiometer) images [54] (product MOD09Q1). We used Google Earth Engine [55, 56], to retrieve the NDVI value of the closest date to each GPS fix. We evaluated the information retrieved to ensure all images were of good enough quality to be used in the study [55]. NDVI was calculated as the difference between the near infra-red (NIR) and the red (R) reflectance values over the sum of the two [57]:

$$NDVI=\frac{NIR-R}{NIR+R}$$

As with relative temperature exposure, we quantified the NDVI experienced by each individual relative to the population average by fitting a GAM to model daily NDVI for each individual as a smoothed function of Julian day. We then summed the residuals for each individual as an index of its relative NDVI with respect to the studied population during each individual’s tracking period (hereafter designated as relative NDVI).

The post-breeding period began on the day each bird completed the migration (i.e. reached the post-breeding area) and ended on 15th of September. In this period of the annual cycle the species is exposed to the highest temperatures and to food shortages [38]. The same three environmental variables were calculated: relative temperature exposure, the percentage of available microclimate refugia and relative NDVI, following the method used for the breeding season.

### Statistical analysis

Repeatability (R) is commonly evaluated as the intra-class correlation coefficient (ICC) which reflects the degree of consistency of each individual’s behaviour or response [58]. R varies between 0 and 1, where 0 indicates the same degree of variation in the individual’s repeated behaviour as the variation in the population, and 1 indicates a strong reliability on the individual behaviour or response [59, 60].

We estimated the repeatability of migratory timing (departure and return dates from and to the breeding area) and distance travelled using Generalized Linear Mixed Models (GLMM) and parametric bootstrapping with 1,000 iterations, to estimate the associated uncertainty. All migratory and resident birds were used in the analysis, with the individual ID used as grouping factor and the breeding population as a random effect. The consistency analysis was carried out using the *rpt* function from the “rptR” package [61].

We fitted Linear Mixed-effects Models (LMM) from the *lme4* package [62] to analyse the influence of ecological variables (percentage of microclimate refugia availability, relative temperature exposure and relative NDVI) on migratory departure dates, with individual ID as a random factor.

Unlike departure dates, which were broadly normally distributed, return dates were strongly bimodal (Supplementary material: S1). We therefore converted the return dates to a binary variable (pre- and post-November 30th) [37] and fitted a Generalized Linear Mixed Model (GLMM) [63] with a binomial error distribution and a logit-link function [64] to examine the effects of climatic variables on the probability of early or late return.

The return date model included the climatic and relative NDVI (as a proxy for food availability) variables for both the preceding breeding and the post-breeding seasons (until 15th of September) to account for potential seasonal carry-over effects. The influence of ecological variables on migratory timing was assessed using only migratory birds (i.e., excluding resident individuals). We did not analyse environmental correlates of distance travelled between breeding and post-breeding sites, as the consistency analysis showed that individuals did not vary significantly in migration distance between years, indicating that migration distances are unlikely to be influenced by short-term environmental conditions (see Results section).

Model selection for migratory timings was carried out using the “*MuMIn*” [65] R package. All models within ∆AICc < 2 of the top model were considered plausible and thus presented separately (Akaike’s Information Criterion corrected for small sample size) [66, 67].

For the return date model, due to the low sample size [68], we limited the number of variables included in each model to three, and tested all three-way combinations of all variables.

To evaluate potential spatial autocorrelation, we used spline correlogram plots with 95% pointwise confidence intervals calculated using 500 bootstrap resamples [69, 70]. These spline correlograms were run using model residuals, after any spatial autocorrelation explained by the explanatory variables had been accounted for [69, 70]. Spline correlograms were produced using the “*ncf*” R package [71].

We tested for multicollinearity between variables, aiming for − 0.7 > r < 0.7 and a variance inflation factor (VIF) smaller than 3 [64]. All models and summary statistics were run in the R version 4.1.0 [50].

## Results

### Migration timing and patterns

**Fig. 1:**
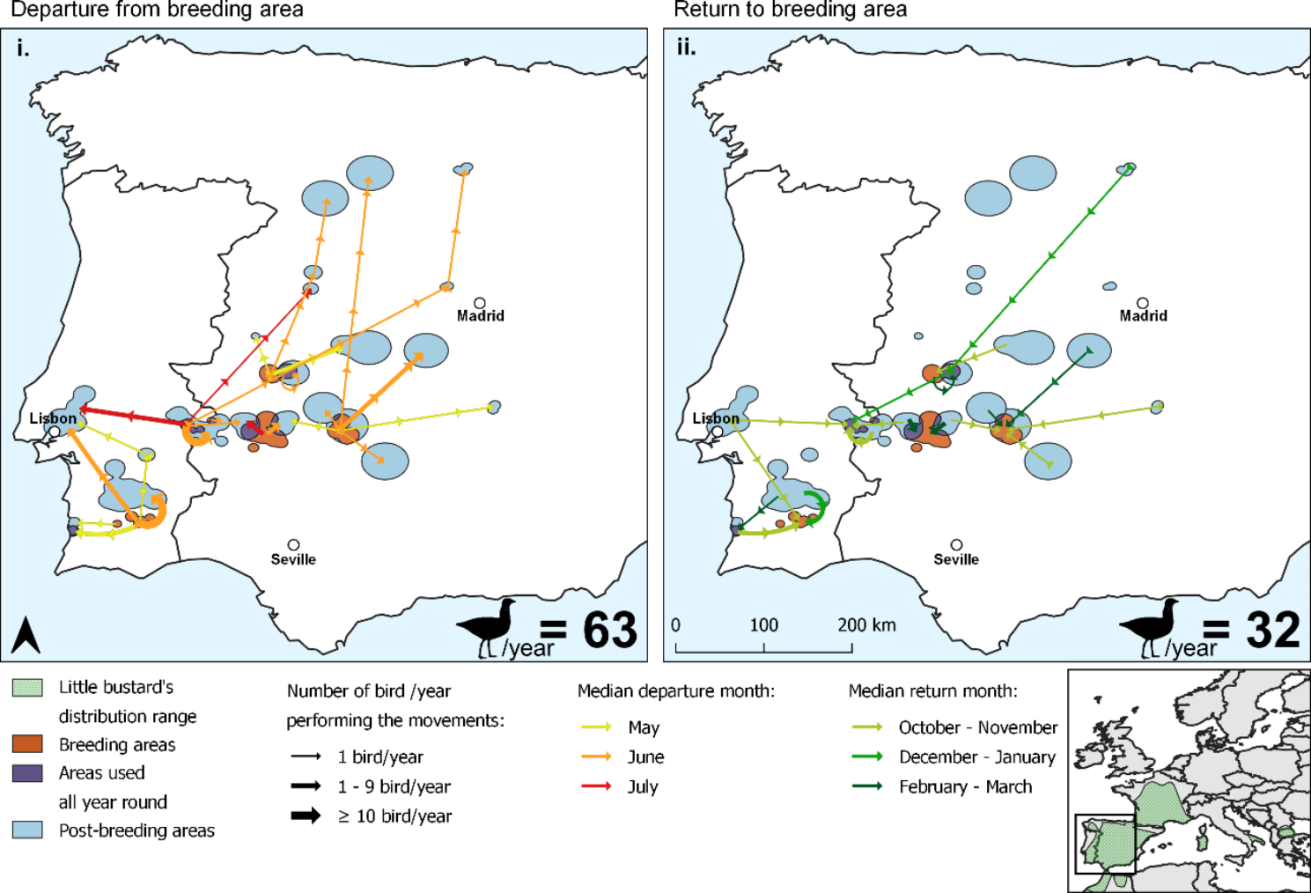
Migration movements of little bustards from breeding (orange) to post-breeding (blue) areas, revealed from GPS tracking data obtained in the Iberia Peninsula. **i** All 63 breeding to post-breeding movements; **ii** All 32 post-breeding to breeding movements. The areas used all year round are represented in purple. The arrows represent the movements to and from post-breeding areas, each colour representing a different month. The little bustard’s distribution [36] is represented in green on the inset map

Of the 67 post-breeding movement events, 63 departed the breeding area between the 10th of May and the 22nd of August, with most birds leaving their breeding grounds during June (median = 20th June ± 23 days) (Fig. 1). One bird switched from migration to residency, remaining in the post-breeding area in the second year (southwestern area, Fig. 1). The remaining three individuals/years adopted a resident strategy, remaining in the same area throughout the year (moving less than 1.5 km from the breeding area).

The distance travelled varied greatly between birds, with movements ranging from 4 to 421 km, and with some birds using more than one post-breeding area (Fig. 1). Some of the breeding areas were also used as post-breeding areas, either by resident birds, birds with short migratory movements, or birds that moved from other breeding areas (shown in purple in Fig. 1).

Out of the 63 migratory events, we were able to collect 32 return migratory movements. Contrary to the departure movement, the return migration was usually direct between the post-breeding and breeding areas. These movements occurred across an extended period of the year, between the 24th of September and 25th of April, with most return movements occurring between October and November (median = 29th November ± 68 days) (Fig. 1).

### Individual migratory consistency

**Fig. 2:**
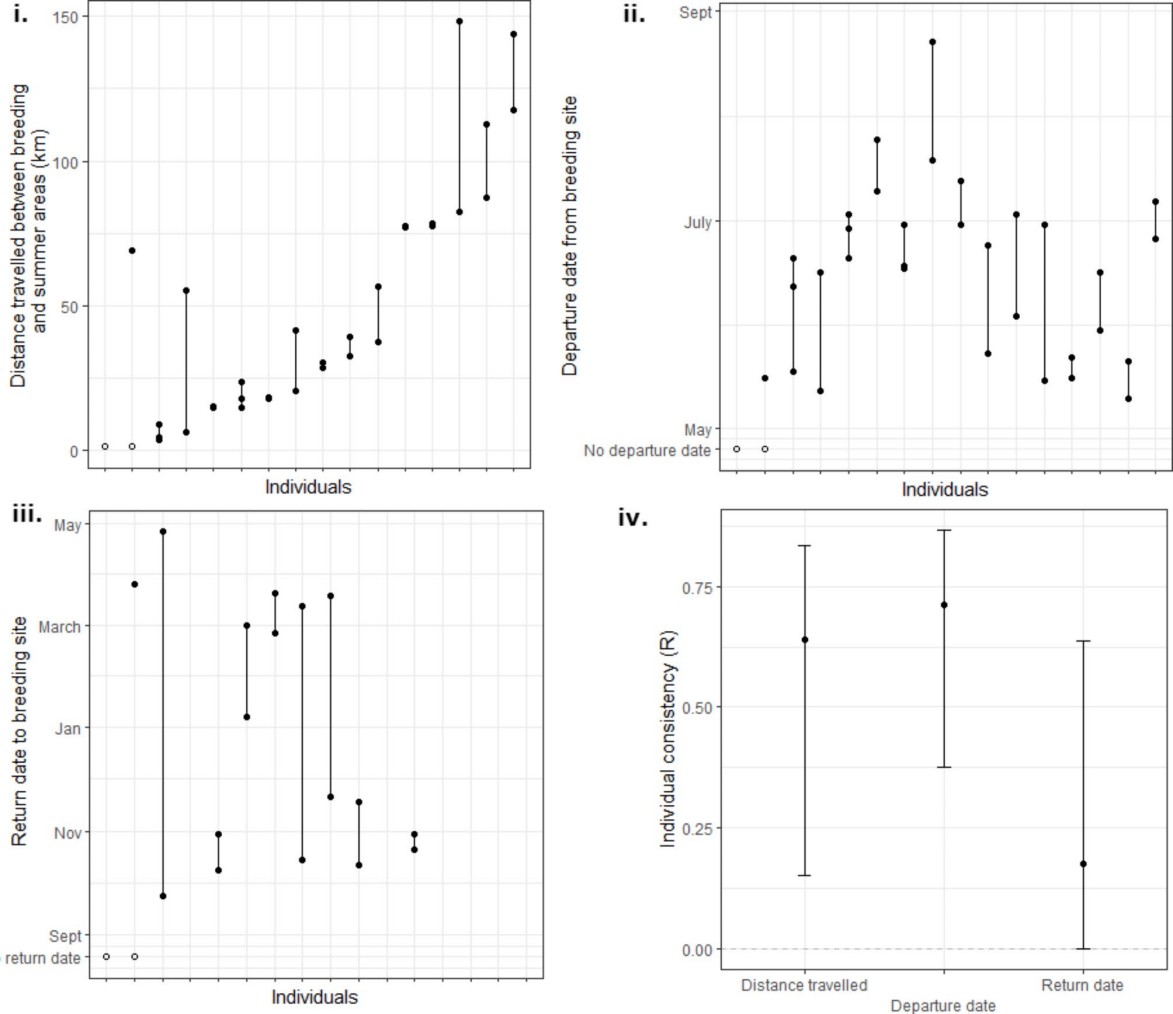
Values of three phenological behaviours: distance travelled (**i**), departure date (**ii**) and return date (**iii**), during multiple years of male little bustards (n = 36 birds). Values for the same individual are linked by vertical lines. Resident birds are shown as white dots, with no departure or return dates. **iv** Individual consistency (R) of the three migratory related variables showing the total population level variance, explained by consistent, repeated individual behaviour. Estimated repeatability does not differ significantly from zero, where the 95% CI bar overlaps with R = 0 (grey dotted horizontal line)

Individuals showed significant repeatability between years in the distance travelled between breeding and post-breeding areas (R = 0.64, Fig. 2i–iv, Supplementary material: S2), suggesting that individuals are unlikely to vary their migration distance in response to environmental conditions. However, dates of departure from and to the breeding area did not show significant repeatability between years (R = 0.35 and R = 0.17, respectively, Fig. 2ii–iv, Supplementary material: S2), suggesting that migration timings could vary with respect to environmental conditions experienced by individuals.

### Departure date in relation to climatic conditions

**Fig. 3:**
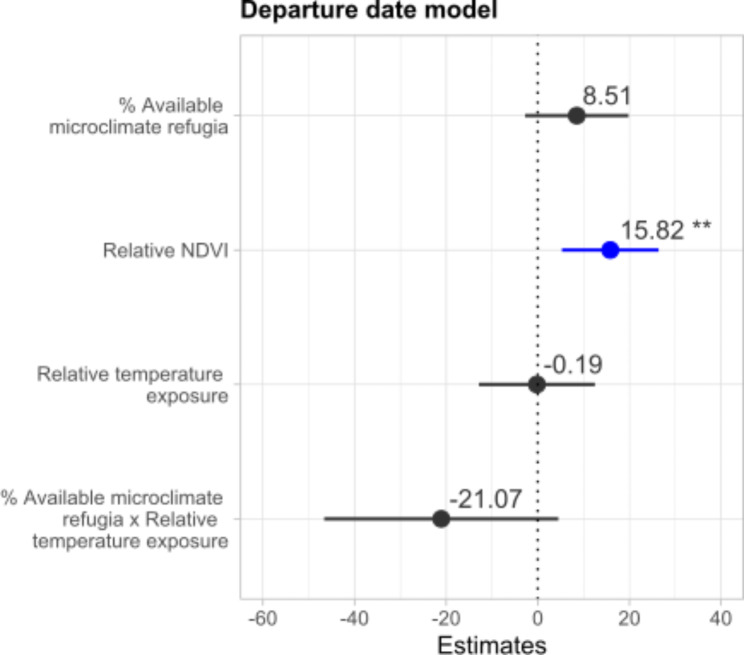
GLMM results for the predictors of departure date from the breeding areas. Variable significancy is shown: ** p < 0.01; * 0.01 < p < 0.05; others, p > 0.05. Positive effects are shown in blue, negative effects in red and not significant effects in black

**Fig. 4:**
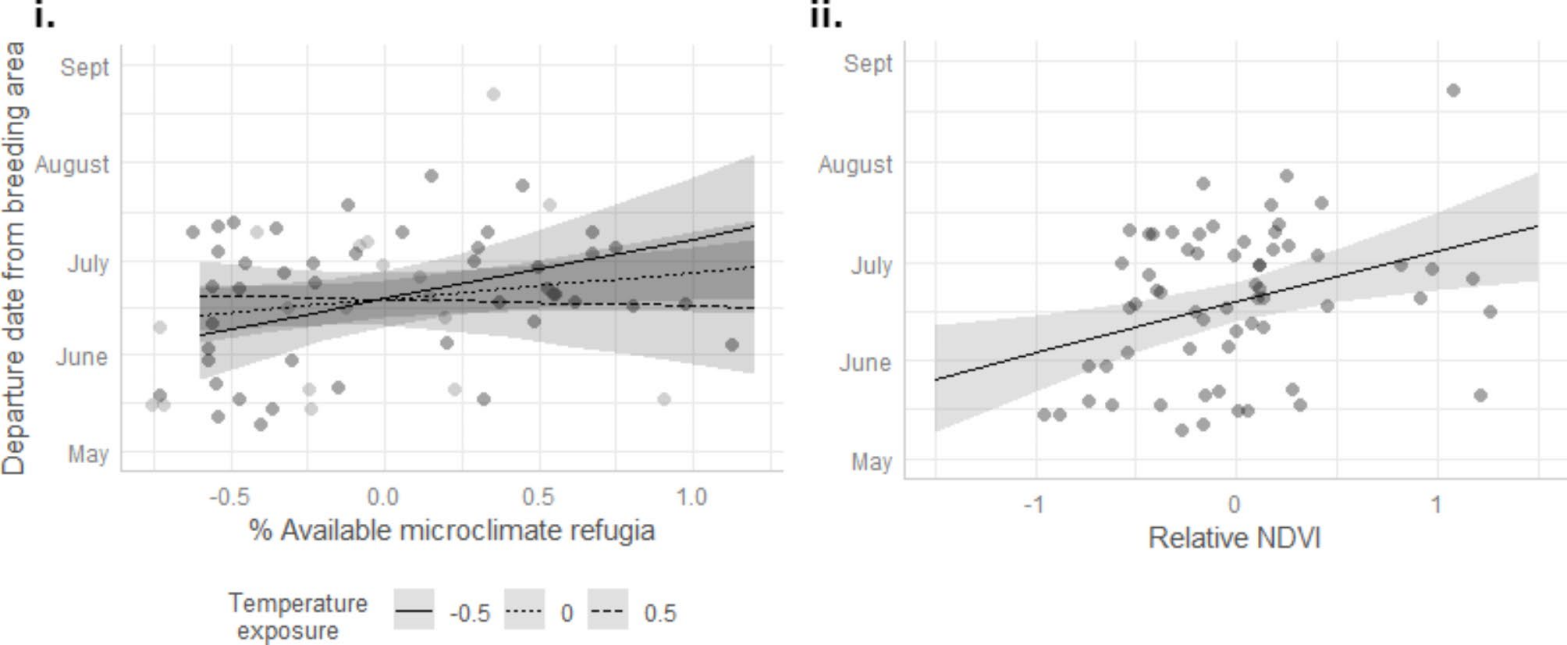
Relationship between the breeding area departure date and **i** relative NDVI and **ii** the interaction between the percentage of microclimate refugia and temperature exposure

The top model explaining variation in individual departure dates included effects of the percentage of available microclimate refugia, relative temperature exposure and their interaction, and relative NDVI. There was some support for the positive effect of percentage of available microclimate refugia during the breeding season on departure date as it was retained in the top model set, although the coefficient was not significant (F = 8.51, p = 0.131). There was strong support for a significant positive relation between departure date and relative NDVI (F = 15.82, p = 0.003) (Figs. 3 and 4i). There was also some support for a marginally significant interaction between available microclimate refugia and relative temperature exposure, such that the positive effect of microclimate refugia on departure date is reduced at higher temperature exposure levels (F = 21.07, p = 0.099) (Figs. 3 and 4ii).

### Return date in relation to climatic conditions

Neither the relative temperature, microclimate refugia, nor NDVI during the breeding or postbreeding seasons showed any significant relationship with variation in individual return dates, with the null model being identified as the most parsimonious model (Supplementary material: S3).

## Discussion

Our results show that the migratory timing of male little bustards is influenced by environmental variables measured at the fine spatiotemporal scales experienced by individuals. While the distance travelled after the breeding season was consistent from one year to the next within individuals and is thus likely to be strongly influenced by site fidelity [72], the departure date varied with food availability (as indicated by relative NDVI). Interestingly, we also found some support that birds inhabiting sites with greater refugia availability also left their breeding sites later, though this effect was statistically uncertain and reduced when individuals were exposed to the highest temperatures. Return dates to the breeding area were highly variable; we could not detect any relationship with environmental cues during the breeding or post-breeding seasons, suggesting other factors may influence the timing of pre-breeding movements.

### Migration timing and patterns

Partial migration in the Iberian Peninsula’s little bustard population has previously been shown to be associated with resource depletion in the breeding sites and extreme temperatures during post-breeding [37, 38]. We found a high degree of variability in timing of little bustards’ post-breeding movements, with birds moving to the post-breeding areas between May and the end of August, with most movements occurring during June. This extended migration period has previously been associated to the different migratory strategies within the Iberian population [37]. Our tracked birds mainly moved north, or to coastal or higher-altitude areas. In these areas, individuals may encounter lower temperatures and higher food availability than the breeding sites during the non-breeding period [37, 38].

Residents, while in low numbers (only three birds, followed for four seasons in total), were detected at multiple breeding populations. Iberian little bustards were historically described as resident/sedentary birds [73], but this is now thought to be a less frequent strategy [37]. Most little bustards are short to medium-distance migratory birds and their behaviour is most likely a genetic trait [74], even though other non-genetic factors, such as environmental conditions and individual fitness, may influence this behaviour [75]. One bird in our study shifted from a migratory to resident behaviour over the course of the two years of tracking. This bird remained in its first tracked post-breeding area for the subsequent breeding and post-breeding seasons. As previously shown, species with greater variability in their migratory strategies tend to be more resilient to environmental change. Hence, partial-migration can potentially increase species resilience and adaptation to changing environments [12]. The return dates varied greatly over a period of seven months, with birds returning to the breeding sites between the end of summer (September) and the start of the breeding season (April).

### Individual migratory consistency

Like other bustard species, male little bustards show high breeding site fidelity and postbreeding site fidelity [72, 76]. If no major habitat changes occur, there is a high probability of birds using the same breeding and post-breeding sites over multiple years [37] as shown by the high repeatability of distances travelled between breeding and post-breeding sites found in this study. Postbreeding sites are likely to be selected during the bird’s first migratory attempt [76]. Understanding the environmental cues that influence the first migration is thus likely to be critical in determining the drivers of variation in migration distance. Juvenile tracking is, therefore, an important priority for future studies.

While most of our tracked birds showed high consistency in the distance travelled, some individuals changed post-breeding sites between years (Fig. 2i). We hypothesise that these differences could reflect changes in habitat between years or extreme climatic events, such as drought years, which may lead birds to relocate to areas with higher post-breeding productivity. Changes could also relate to the age of the individual, with older, more experienced birds having more consistent migratory routes [1, 77, 78]. Additional multi-year tracking is needed to test these hypotheses.

The timing of migration of different migratory bird species has previously shown to be associated with climate variables measured at coarse scales, including temperature, precipitation, and wind [16]. In our study, both the departure and return dates to breeding areas showed lower within- than between-individual consistency, suggesting a potential influence of environmental factors on these dates.

### Departure date in relation to environmental conditions

We also found some support that males within areas with greater availability of microclimate refugia were more likely to leave their breeding grounds later, although this effect was only marginally significant. Microclimate refugia occur in areas with greater heterogeneous thermal landscapes, promoted by the existence of small patches of non-herbaceous vegetation (trees and shrubs) [26, 79, 80]. The interaction between refugia availability and temperature, which was marginally significant (p = 0.099), suggested that individuals exposed to very high relative temperatures may depart from the breeding area earlier, regardless of the availability of microclimate refugia. This is possibly related to a thermal limit, above which the available microclimate refugia within the region can no longer buffer individuals against thermal stress. Nevertheless, the positive effect (although not significant) of microclimate refugia availability at medium and lower temperature exposure levels suggests the potential importance of microclimate refugia in prolonging the breeding season in this species.

Temperatures experienced by individuals, although included in the model, had no significant linear effect (F = -0.19) in influencing their departure date from breeding areas. However, previous studies showed that temperature can be a critical factor in movement phenology [13, 14, 16]. Additionally, little bustards are known to reduce their activity at temperatures above 25 °C [45]. We hypothesise that this lack of significant effect of temperature may be due to exposure to high temperatures throughout the breeding season, expected due to recent warming (see Ramos et al. 2023 [26]). Exposure to high temperatures may affect the breeding and feeding behaviour hence may not be as strongly associated with the timing of movement.

We found a positive influence of relative NDVI on post-breeding departure dates. NDVI is considered a good proxy for assessing food availability for this herbivorous species [53], and food availability is known to be a key determinant of habitat quality for grassland birds [38, 81, 82]. Additionally, NDVI is correlated to precipitation and temperature [83], two climatic variables known to influence migration timings [16]. In the Iberian Peninsula, NDVI peaks during April/May and decreases steeply between the end of May and June, as the ambient temperature increases [84]. As a result, areas with higher NDVI levels later in the breeding season are likely to support breeding for a longer period of time.

Although not included in this analysis, other studies have pointed to wind as a crucial factor in determining migration timings, alongside precipitation and temperature [16]. Since most individuals migrate short distances at low altitudes [72] and have an active flapping flight, the use of wind is likely less relevant for this species, while precipitation is a rare event during Mediterranean summers.

### Return date in relation to climatic conditions

Despite the high variability of return dates in our study (Fig. 2 ii and iv), we found no relationship between return dates and any of the environmental variables considered. Although return dates range from September to April, male little bustards do not start displaying until late March/April [85], suggesting that factors unrelated to the timing of breeding influence the return dates. It is possible that the lack of significant relationships was due to low sample size or lack of information about other variables, such as grazing regimes, vegetation height, and land cover type, that greatly affect little bustards’ post-breeding habitat selection [38, 86]. Moreover, disturbance (human and livestock) can force the birds to change areas, including returning to the breeding site earlier [87]. Much attention has been given to the effects of climate change on return (pre-breeding) migration, mainly for long-distance migrants [88, 89], and less attention is given to climatic features influencing post-breeding migration. Our findings suggest that in this species, climate variables (particularly temperature) are more important in determining the timing of departure from breeding area (autumn migration) than the return (winter/spring migration) dates.

### Conservation implications

Understanding how different migratory strategies are maintained in a population is crucial, especially for declining species where the presence of diverse movement strategies can help promote resilience to environmental change [12]. Additionally, exploring the influence that microclimate has in maintaining these strategies can be particularly relevant when designing conservation measures to enhance the availability of climate refugia across landscapes.

Microrefugia are widely recognised for potentially playing a critical role in promoting resilience to climate change, buffering individuals from detrimental environmental conditions, by providing shelter from elevated temperatures, and reducing the energetic costs of thermoregulation [79, 90]. Our study is the first to suggest that microclimate refugia could also extend the breeding season length in a migratory species, suggesting positive impacts on breeding success may occur, by allowing males to stay longer at the lekking areas.

Our results, therefore, potentially could have important potential implications for the design of climate-adaptive conservation measures. With increasing temperatures and lower annual precipitation [34], vegetation in our study region is likely to dry sooner and faster in the future, while temperature exposure will increase. These conditions can lead male little bustards to leave the breeding site early, shortening the breeding period. Provision of habitat features that ensure microclimate refugia (i.e. shrubby herbaceous scattered patches) could increase the availability of areas where birds can thermoregulate at lower metabolic cost during the warmest hours of the day, potentially enabling them to extend their breeding season long enough to maintain viable breeding populations [23].

This study shows climate may play a significant role in determining the end of the breeding season of male little bustards and provides some evidence for how management can potentially extend it, by creating microclimate refugia. This would, ultimately, keep the breeding areas suitable for longer and could play an important role within vulnerable ecosystems to climate change [34].

Although this study focuses on males’ migratory behaviour, our findings likely extend to females, despite having a more restricted post-breeding migratory behaviour, since they singly raise the chicks and carry out late migratory movements [91]. Prolonged stays at the breeding grounds can potentially make them vulnerable to high temperatures and low food availability during the hottest period of the year. Thus, microclimate refugia can potentially, be critically important for females and chicks. Return migration occurs when birds are flocking, and the movements of tagged males are representative of the movements of many individuals. Future studies examining female migratory responses to climate are urgently needed.

## Conclusion

We show that distance travelled varies little within individuals, probably due to their breeding and post-breeding site fidelity, but the timings of migratory movements can vary markedly from year to year. Departure timing from the breeding area was strongly affected by NDVI (a proxy for food availability), and potentially also by microclimate refugia availability, as this variable was included in the best model. Our findings suggest the potential importance of fine-scale habitat features that can act as microclimate refugia, in this case effectively prolonging the stay at breeding grounds in all but the hottest conditions. In our study region, microclimate refugia occur in areas with small patches of non-herbaceous vegetation (trees and shrubs) [26, 79, 80]. Thus, while the presence of open grassland habitat is a critical requirement for little bustards, the existence of small and scattered patches of trees and shrubs may play an increasingly important role in determining habitat quality for this species in a warming world.

### Electronic supplementary material

Below is the link to the electronic supplementary material.

Supplementary Material 1

## Data Availability

The datasets used and/or analysed during the current study are available from the corresponding author on reasonable request.
