# Supplementary material for: Temperature and microclimate refugia use influence migratory timings of a threatened grassland bird
Source: Mov Ecol. 2023 Dec 1;11:75. doi: 10.1186/s40462-023-00437-7 (PMC10691164; doi:10.1186/s40462-023-00437-7)
Supplement: Supplementary file 1 — Supplementary Material 1 [file 40462_2023_437_MOESM1_ESM.docx]

**Temperature and microclimate refugia use influence migratory timings of a threatened grassland bird**

Rita F. Ramos, Aldina M.A. Franco, James J. Gilroy, João P. Silva

**S1 – Return dates distribution**


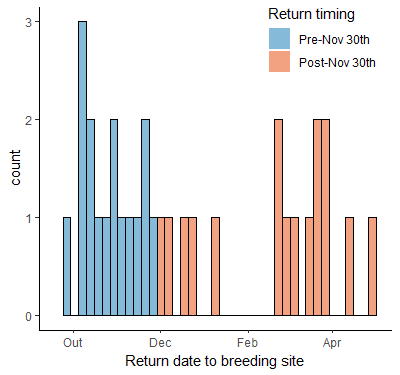


**Figure S1 –** Histogram for the return dates of little bustards to the breeding site (i.e. pre-breeding migration), colour-coded as the binary variable (pre- and post-November 30th) (Garcia La Morena et al. 2015)

**S2 – Individual migratory consistency**

| **i.**  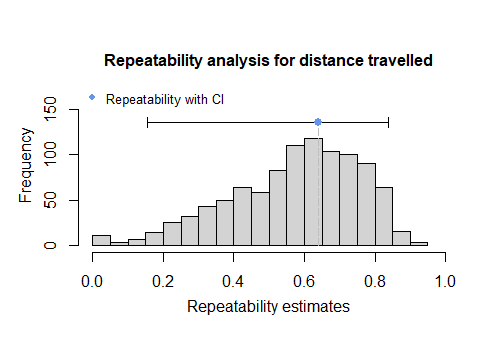 | **ii.**  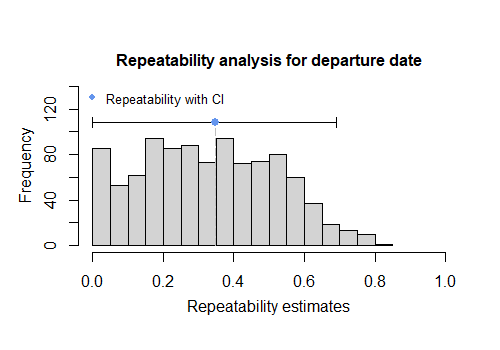 |
| --- | --- |
| **iii.**  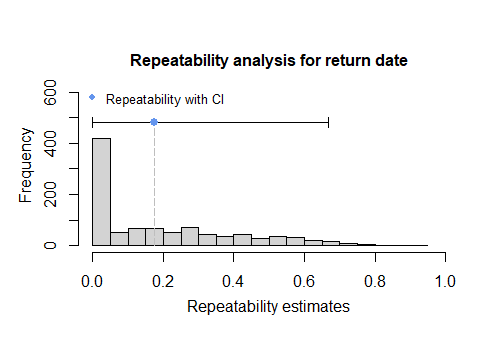 |  |

**Figure S2 –** Plots of the distribution of the parametric bootstrap samples, point estimate and the limits of the confidence interval for i. distance travelled; ii. departure date and iii. return date between the breeding and post-breeding areas

**S3 – GLMM summary for the candidate models from MuMin for the predictors of return date**

**Table S3 -** GLMM summary for the top Δ AICc < 2 predictors of return date to the breeding areas. Due to the low sample size, two distinct models were runed, using the temperature, microclimate refugia availability and NDVI conditions during the breeding season (breeding variables) and using the same variables during the post-breeding season (post-breeding variables). In all cases the null model was selected, and being the most parsimonious one, we can not explain the variables responsible for the return dates to the breeding area

| Model | Variables | | | | | AICc | Δ AICc |
| --- | --- | --- | --- | --- | --- | --- | --- |
|  | *Intercept* | *Microclimate refugia availability* | *Relative temp. exposure* | *Interaction term:*  *microclimate refugia availability vs.*  *relative temperature exposure* | *NDVI* |  |  |
| *Using breeding variables* | | | | | | | |
| *1* | 0.012 |  |  |  |  | 48.60 | - |
| *2* | 0.007 |  |  |  | -0.623 | 50.46 | 1.86 |
| *Using post-breeding variables* | | | | | | | |
| *1* | 0.012 |  |  |  |  | 48.60 | - |
| *2* | 0.012 |  |  |  | 0.896 | 49.83 | 1.23 |
| *3* | 0.012 |  | 0.594 |  |  | 50.50 | 1.90 |

**References**

Garcia De La Morena EL, Morales MB, Bota G, Silva JP, Ponjoan A, Suárez F, et al. Migration patterns of Iberian little bustards *Tetrax tetrax*. Ardeola. 2015;62:95–112.
